# A White Paper on Collagen Hydrolyzates and Ultrahydrolyzates: Potential Supplements to Support Joint Health in Osteoarthritis?

**DOI:** 10.1007/s11926-021-01042-6

**Published:** 2021-10-30

**Authors:** Ali Mobasheri, Armaghan Mahmoudian, Ursule Kalvaityte, Ilona Uzieliene, Christina E. Larder, Michèle M. Iskandar, Stan Kubow, Paulo Cesar Hamdan, Cyro Scala de Almeida, Lacey J. Favazzo, Luc J.C. van Loon, Pieter J. Emans, Pérola G. Plapler, Michael J. Zuscik

**Affiliations:** 1 Research Unit of Medical Imaging, Physics and Technology, Faculty of Medicine, University of Oulu, Oulu, Finland; 2 Department of Regenerative Medicine, State Research Institute, Centre for Innovative Medicine, Vilnius, Lithuania; 3 Departments of Orthopedics, Rheumatology and Clinical Immunology, University Medical Center Utrecht, Utrecht, The Netherlands; 4 Department of Joint Surgery, The First Affiliated Hospital of Sun Yat-sen University, Guangzhou, 510080 Guangdong China; 5 World Health Organization Collaborating Center for Public Health Aspects of Musculoskeletal Health and Aging, Université de Liège, Liège, Belgium; 6 Department of Clinical Sciences Lund, Orthopaedics, and Skeletal Biology, Clinical Epidemiology Unit, Lund University, Lund, Sweden; 7 School of Human Nutrition, McGill University, 21,111 Lakeshore, Ste. Anne de Bellevue, QC, H9X 3V9 Canada; 8 Hospital Universitário Clementino Fraga Filho, Department of Traumatolgy and Orthopedics of Medical Faculty of Universidade Federal do Rio de Janeiro, Rio de Janeiro, RJ Brazil; 9 Santa Casa Sao Paulo, Sao Paulo, SP Brazil; 10 Colorado Program for Musculoskeletal Research, Department of Orthopedics, University of Colorado Anschutz Medical Campus, Aurora, CO USA; 11 Department of Human Biology, NUTRIM School of Nutrition and Translational Research in Metabolism, Maastricht University Medical Centre+, Maastricht, The Netherlands; 12 Department of Orthopaedic Surgery, CAPHRI School for Public Health and Primary Care, Maastricht University Medical Centre, Maastricht, The Netherlands; 13 Divisão de Medicina Física, Instituto de Ortopedia e Traumatologia do Hospital das Clinicas da Faculdade de Medicina da, Universidade de São Paulo (FMUSP), São Paulo, SP Brazil

**Keywords:** Joint health, Osteoarthritis, Nutritional supplement, Nutraceutical, Denatured collagen, Collagen hydrolyzate, Collagen ultra-hydrolyzate

## Abstract

**Purpose of Review:**

Osteoarthritis (OA) is the most common forms of arthritis in the general population, accounting for more pain and functional disability than any other musculoskeletal disease. There are currently no approved disease modifying drugs for OA. In the absence of effective pharmacotherapy, many patients with OA turn to nutritional supplements and nutraceuticals, including collagen derivatives. Collagen hydrolyzates and ultrahydrolyzates are terms used to describe collagens that have been broken down into small peptides and amino acids in the presence of collagenases and high pressure.

**Recent Findings:**

This article reviews the relevant literature and serves as a White Paper on collagen hydrolyzates and ultrahydrolyzates as emerging supplements often advertised to support joint health in OA. Collagen hydrolyzates have demonstrated some evidence of efficacy in a handful of small scale clinical trials, but their ability to treat and reverse advanced joint disease remains highly speculative, as is the case for other nutritional supplements.

**Summary:**

The aim of this White Paper is to stimulate research and development of collagen-based supplements for patients with OA and other musculoskeletal diseases at academic and industrial levels. This White Paper does not make any treatment recommendations for OA patients in the clinical context, but simply aims to highlight opportunities for scientific innovation and interdisciplinary collaboration, which are crucial for the development of novel products and nutritional interventions based on the best available and published evidence.

## Introduction

OA is believed to impact more than 300 million people worldwide [1]. It is estimated that a “tsunami” of new OA cases will hit countries with a much larger aging population in the developed world by the year 2050 [2]. However, these numbers provided by epidemiological studies are likely an underestimation, and the true burden of OA is likely to be much higher as accurate data are not available for sub-Saharan Africa, Central America, or South America. A recent commentary published in The Lancet has proposed that the incidence of OA is much higher, estimated at around 7% of the global population; this means that more than 500 million people worldwide have OA [3].

OA is an especially problematic disease as there are currently no effective pharmacological treatments and no disease modifying OA drugs (DMOADs). There is some correlation between published treatment guidelines overall but there is no clear consensus in any of the treatment guidelines regarding nutraceuticals and supplements. Furthermore, the guidelines and recommendations for the management of OA are difficult to follow for most healthcare professionals and patients, often leaving them dissatisfied and confused. Patients also remain dissatisfied with the currently approved pharmacological interventions; in the absence of DMOADs, they resort to using nutritional supplements and nutraceuticals. Recent guidelines have been published by the American College of Rheumatology (ACR), Arthritis Foundation (AF), the European League Against Rheumatism (EULAR), the European Society for Clinical and Economic Aspects of Osteoporosis, Osteoarthritis and Musculoskeletal Diseases (ESCEO), and Osteoarthritis Research Society International (OARSI) [4–6]. The recent ACR/AF 2020 OA treatment guidelines only focus on management options that are available in the USA and are restricted to pharmacologic therapies and agents that are available in pharmaceutical-grade formulations, thus eliminating consideration for most nutraceuticals according to ACR and FDA criteria [7, 8]. The OARSI 2019 treatment guidelines do not include nutraceutical products because the OARSI expert group has strongly argued that the formulations have not yet been standardized [9]. However, the ESCEO 2019 Treatment Guidelines Working Group continues to advocate for the use “pharmaceutical grade” or “prescription grade” crystalline glucosamine sulfate (GS) and chondroitin sulfate (CS) as step 1 in pharmacological treatment. ESCEO argues that the formulations for GS and CS in Europe are standardized as “pharmaceutical grade” or “prescription grade” [10].

The lack of consensus on supplements creates major challenges for the research community, healthcare professionals, and OA patients, especially those who continue to use supplements combined with over-the-counter (OTC) medications [11]. Individuals with OA using supplements also report using OTC products in combination with prescription products and the likelihood of using prescription products increases with the length of OA history [11]. This suggests that patients continue consuming supplements irrespective of what the treatment guidelines might state.

Another major challenge in the use of nutritional supplements in OA is the use of terminology; vocabulary used to describe supplements is highly variable. Some papers refer to these products as food supplements while others refer to them as nutritional supplements or nutraceuticals. The literature often refers to them as complementary and alternative medicines, and there are papers that refer to plant-derived supplements as botanical and herbal supplements. The phrases “food supplements” and “nutraceuticals” have been used interchangeably since both types of supplements claim to benefit health. It is important to define key terms that have been accepted by regulatory agencies (Table 1).

**Table 1:** Definitions of nutritional supplements and nutraceuticals

**Term**	**Source**	**Definition**
Food supplement	United States Government Office, 1994	A product (other than tobacco) in the form of a capsule, powder, softgel, or gelcap intended to supplement the diet to enhance health that bears or contains one or more of the following dietary ingredients: a vitamin, mineral, amino acid, or other botanical or dietary substance. United States Food and Drug Administration (FDA). Dietary Supplement Health and Education Act (DSHEA). U.S. Department of Health and Human Services. 1994. United States. Public Law 103–417. https://www.fda.gov/food/information-consumers-using-dietary-supplements/questions-and-answers-dietary-supplements
Food supplement	European Union (EU) and European Commission (EC), 2002	Food product whose purpose is to supplement the normal diet and which consists of a concentrated source of nutrients or other substances with nutritional or physiological effects, single or in combination, marketed in dosed formulations, such as capsules, tablets or pills, designed to be taken in small individual quantities measured. EU Directive 2002/46/EC https://ec.europa.eu/food/safety/labelling_nutrition/supplements_en https://eur-lex.europa.eu/eli/dir/2002/46/oj
Nutraceuticals	Brower V., 1998	Any substance that is a food or a part of a food and is able to induce medical and health benefits, including the prevention and treatment of disease [13]
Nutraceuticals	European Nutraceutical Association (ENA), 2016	Nutritional products that provide health and medical benefits, including the prevention and treatment of disease [14]

Nutraceuticals are derived from a food or part of a food that is aimed toward disease prevention or treatment, whereas food supplements are generally referred to as single substances used either alone or in a mixture to support micronutrient needs [12]. In this White Paper, we discuss, among other agents, the potential for using collagen or collagen hydrolyzates as novel and innovative nutraceuticals to support joint health and provide prophylactic treatment for people with OA.

## Opportunities for Management of OA with Nutraceuticals

Nutraceuticals and natural products for OA are sold and marketed for their antioxidative and anti-inflammatory properties, with many manufacturers claiming they possess therapeutic, anabolic, and regenerative effects [15–18]. Recently published data from a small number of studies of herbal and botanical nutraceuticals developed from natural products have provided promising efficacy data compared to placebo comparators, but their potential for treating OA requires further confirmation in larger clinical trials [19••, 20].

Currently, nutraceuticals constitute a wide variety of natural product extracts generated from different plants and animals, as well as their derived active ingredients [4]. Although nutraceuticals have gained enormous popularity in patient-driven inflammatory disease management, detailed mechanistic evidence of their efficacy in OA is still lacking [21].

## Collagen Supplements

### Non-hydrolyzed Collagen

**Fig. 1:**
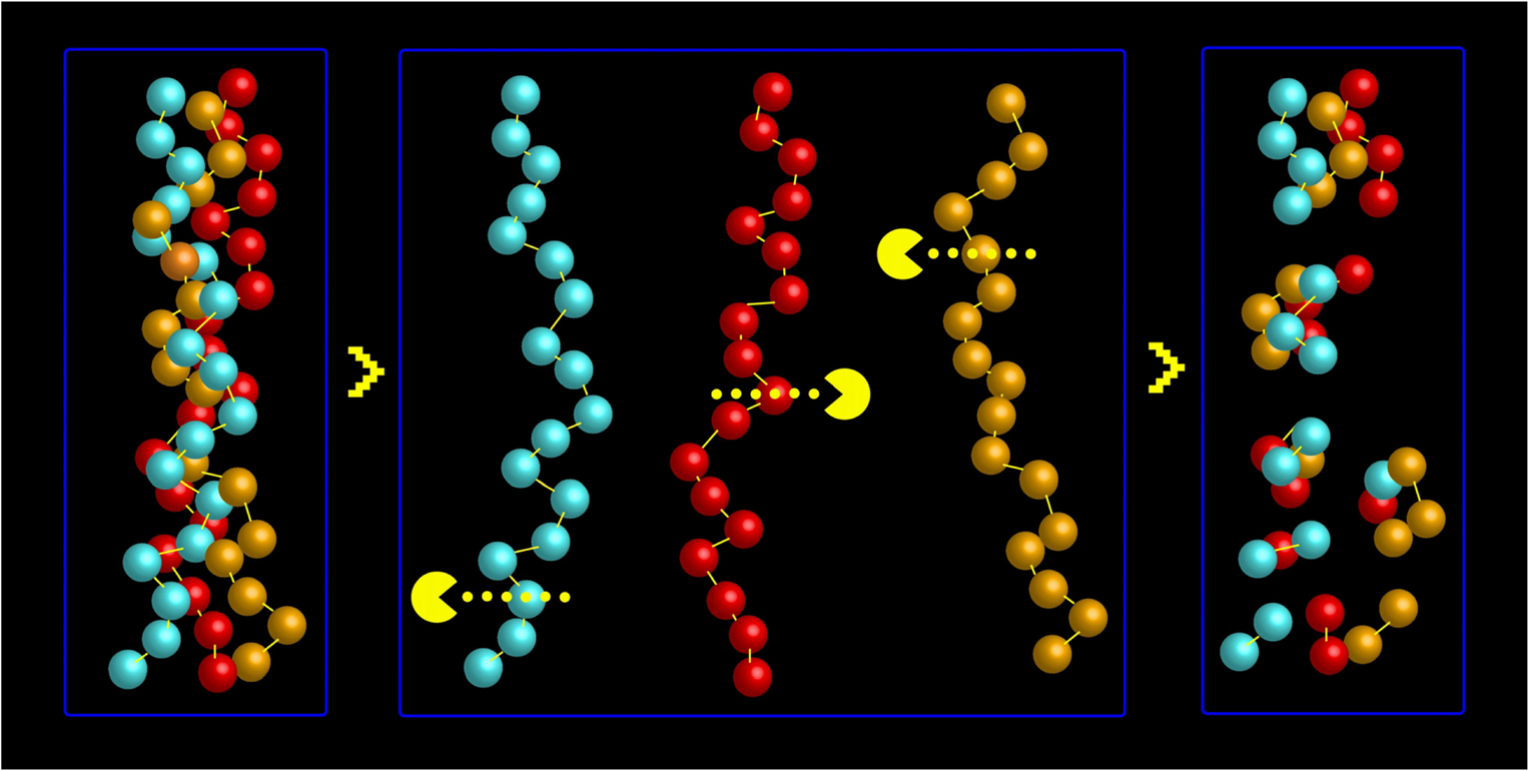
Schematic illustration of the ultrahydrolyzed collagen concept

These nutritional supplements are often a by-product from the food industry. Collagen supplements are rich in amino acids such as glycine, proline, and hydroxyproline; all of which play important roles in the building of joint cartilage and may also have anti-inflammatory and antioxidant effects, and have been speculated to act as signaling molecules [5]. While a seminal study published in Science in 1993 revealed efficacy of oral type II collagen supplementation in reducing joint swelling in RA [6], trials into the role of collagen supplementation in treating OA have demonstrated inconsistent results [19••]. There have been several reported analgesic and anti-inflammatory effects of collagen in unpublished clinical trials, but according to recently published systematic and narrative reviews, these have not been reproduced across studies [19••, 20]. Further trials with improved study designs are therefore needed to evaluate their proposed nutraceutical potential (Fig. 1).

**Fig. 2:**
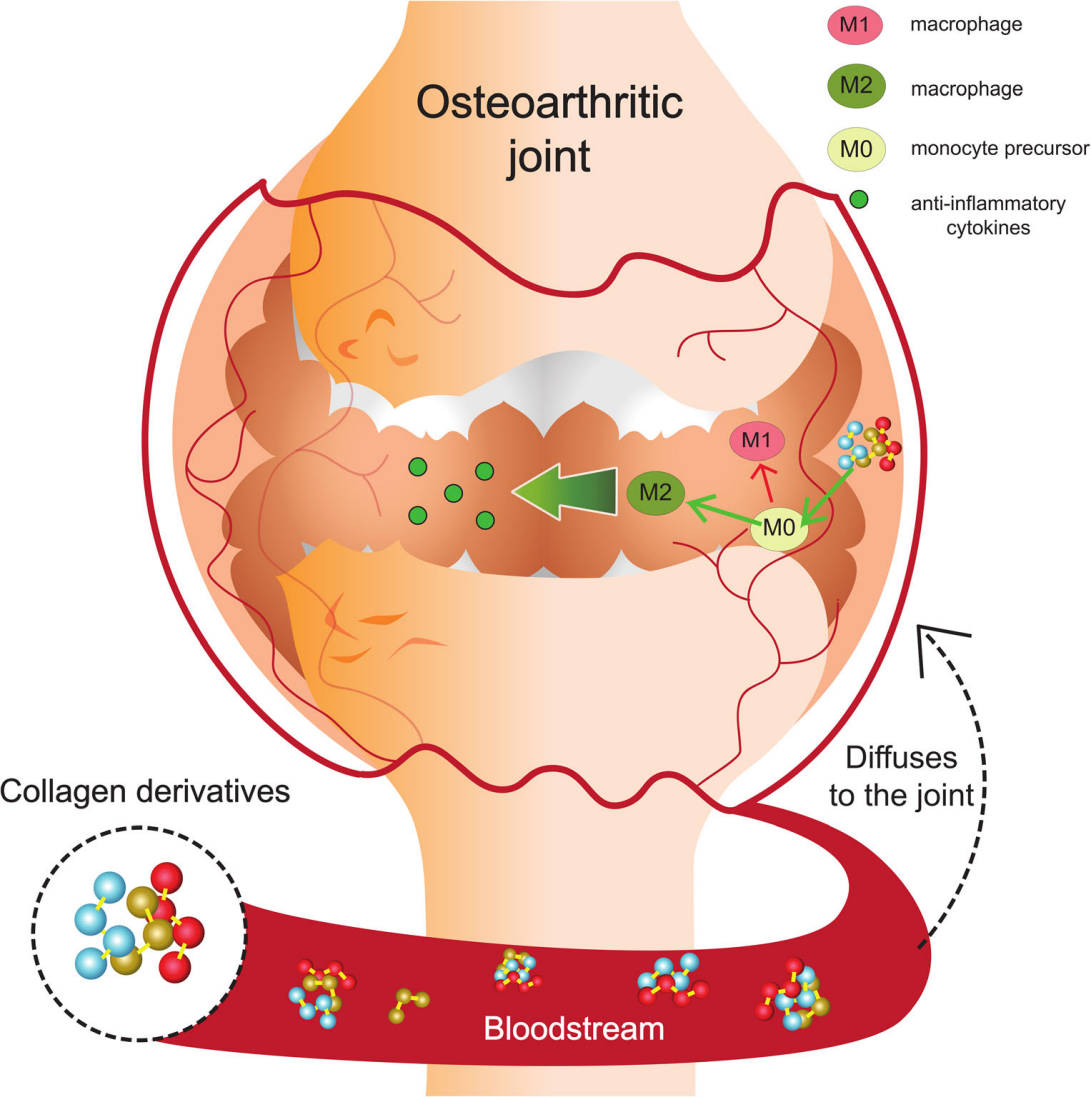
Proposed concept for the delivery of collagen-derived peptides to the synovial joint. Possible mechanism for immunometabolic and phenotypic reprogramming of macrophages by peptides derived from ultrahydrolyzed and hydrolyzed collagen

Nutraceutical supplements derived from collagen can be made from beef, pork, or fish bones and skins, which undergo processing to increase the bioavailability of their amino acids and/or peptides; the enzymatic hydrolysis of collagens enhances the postprandial absorption of its processed components [22]. Processed and pre-digested collagen products are called collagen hydrolyzates CHs and are sold in the form of collagen capsules at pharmacies and health food suppliers. Different processing and manufacturing methods to make collagen hydrolyzates can yield different products, with differences in amino acid content and peptide sequences that vary in molecular weight (MW). Lower MW peptides may be more easily absorbed in the small intestine, increasing the likelihood of delivery to other areas in the body such as joints (Fig. 2).

Type II collagen is the most abundant protein found in articular cartilage and intervertebral discs. Because type II collagen is the main protein in cartilage, there have been suggestions that oral collagen supplementation may help to support cartilage repair. However, definitive proof for this is still lacking. Different formulations of collagen have been developed based on the degree of hydrolysis, the most prevalent being undenatured collagen and hydrolyzed collagen.

### Undenatured Collagen

Undenatured type II collagen (UC-II) is a patented agent that is often derived from chicken cartilage and has been used in various clinical trials in humans and companion animals, including dogs and horses [23–25, 26•, 27•]. Undenatured collagen is biochemically modified (glycosylated), has a good safety profile [28], and has been speculated, but not proven, to possess immunomodulatory properties. To what extent undenatured collagen is digested and absorbed following ingestion in vivo in humans remains to be assessed, but it has been speculated that potential bioactive peptides may be preserved and absorbed as free amino acids, especially glycine and proline. These amino acids represent quantitatively important precursors for the synthesis of cartilage extracellular matrix (ECM) macromolecules.

Whether UC-II, glycosylated or biochemically modified by some other means, has functional benefits beyond the provision of relevant amino acids as precursors for de novo collagen protein synthesis, will be an important area of research and innovation and exemplifies an opportunity for further evidence-based product development by consumer health companies. Work in this space will need to systematically correlate efficacy at reducing symptoms with mechanism of action, with adaptive trial designs and with many open questions remaining on the ability of UC-II to exert direct effects on cartilage metabolism in joints.

### Hydrolyzed Collagen

Hydrolyzed collagen is a form of collagen that is also referred to as collagen hydrolyzate. Collagen hydrolyzate and gelatin may be the same in terms of amino acid composition, but they possess different chemical properties. Collagen is a native protein molecule with a molecular weight of ~300 kDa [29]; and collagen hydrolyzates are processed intensively to break up the large collagen molecules into smaller fragments to increase absorption (Fig. 1).

To produce hydrolyzed collagen, native collagen undergoes denaturation followed by a hydrolysis process, resulting in very low molecular mass (3–6 kDa) collagen peptides, compared to native collagen size (285–300 kDa) [29]. Different processing and post-processing methods to make collagen hydrolyzates can yield vastly different products, creating different collagen peptide sequences and molecular weights. These differences can potentially impact biological function in terms of regulating joint inflammation and effect on subchondral bone. Furthermore, lower molecular weight collagen peptides may be more easily absorbed in the small intestine, theoretically increasing the likelihood of being delivered to other areas in the body including joints. The resistance of collagen peptides to hydrolysis and digestion is primarily based on amino acid composition. In that regard, peptides with the amino acid proline or hydroxyproline are not readily hydrolyzed, or digested by the gastrointestinal system which may allow them to be absorbed in the small intestine. In support, peptides such as Pro-Hyp and Pro-Hyp-Gly, derived from the repeating motif Pro-Hyp-Gly, have been reported to circulate in the blood up to 4 h after oral collagen and gelatin ingestion [30, 31]. Thus far, there are no published studies that have conducted a quantitative assessment of the actual quantity of collagen derived peptides that are absorbed in the gastrointestinal tract and/or released in the circulation.

A number of clinical trials have been conducted concerning oral supplementation of collagen and its derivatives, undenatured and hydrolyzed collagen. All have shown to be safe and tolerable for the patient, causing no or only mild adverse effects to some patients [32, 33]. One of the concerns regarding oral collagen supplementation is associated with oral tolerance. Oral tolerance is the ability of orally administered antigen to suppress or minimize the immune response, and has been used to manage the occurrence of immunogenicity in other disease areas [34–39].

As for oral tolerance associated with collagen, the response relies on structural properties of the collagen derivative because only specific epitopes found in an intact helix structure of the undenatured collagen are recognized by the immune system. The epitopes interact with gut-associated lymphoid tissue (GALT) and result in reduction of systemic T cell attack on the cartilage as well as reduced joint inflammation and cartilage damage [40–42]. This suggests that, if taken orally, hydrolyzed collagen is digested and broken down into small peptides and amino acids, thus potentially eliminating its immunomodulatory properties.

Due to its lower molecular weight, hydrolyzed collagen has been proposed to have higher bioavailability and solubility, and thus better absorption from the small intestine compared to undenatured collagen [22, 29]. Absorption of orally administered hydrolyzed collagen has been evaluated by studying vascular-perfused rat intestine in situ. The results implied that the breakdown products of hydrolyzed collagen digestion can be absorbed as small peptides [43]. Defining and understanding the difference between collagen products, collagen hydrolyzates, and ultrahydrolyzed collagen can be difficult, confusing, and remains unclear in the literature.

Studies evaluating collagen digestion and amino acid and/or peptide absorption in vivo in humans are required to address the proposed differences in the postprandial bioavailability of collagen-derived amino acids and peptides.

In vitro studies have been used to suggest that collagen derived peptides may: (a) potentially accumulate in cartilage (if given in sufficiently high doses); (b) stimulate chondrocytes to synthesize ECM macromolecules in vitro; and (c) increase osteoblast activity as well as decrease osteoclastic activity [44•, 45, 46–48]. However, whether such peptides are actually absorbed and released in an in vivo setting remains highly speculative. Further studies are required to identify such absorbable bioactive peptides derived from (hydrolyzed) collagen digestion, as the impact of digestion and first pass metabolism on the generation of bioactive peptides from collagen hydrolyzates remains to be investigated. Such research may help to identify peptides and amino acids contributing to the proposed antioxidant and anti-inflammatory properties of collagen hydrolyzate supplementation. In this regard, preclinical research using innovative simulated digestion models in combination with relevant cell/tissue cultures can become an innovative higher throughput platform for investigating new collagen hydrolyzate formulations.

The early papers that appeared on collagen hydrolyzates reported efficacy in the preclinical context (beneficial effects on cartilage metabolism) and improvements in joint pain in the clinical context. Bello and Oesser reviewed the available literature on collagen supplements without date limits and published their results in 2006 [44•]. In addition to published papers, they included abstracts presented at scientific congresses and articles published in German medical journals [44•]. They reported that orally administered collagen hydrolyzate end products can be taken up by the intestine and accumulate in cartilage [44•]. They also proposed that collagen hydrolyzate ingestion can stimulate the synthesis of ECM macromolecules. Bello and Oesser identified four open-label and three double-blind studies. Although some of these clinical trials were of very low quality, they reported that collagen hydrolyzates are safe and may improve pain and function in men and women with OA and other arthritic conditions. It is important to note, however, that the authors included other joint diseases in their review and their focus was not exclusively on OA.

In a trial investigating hydrolyzed collagen and green tea extract supplementation in dogs, the combined treatment decreased indicators of pain in dogs that received the combination product for 3 months. However, the biomarkers selected for evaluation of the effects of supplementation (Coll2–1 and Coll2–1 NO2) were unaffected [49].

An equine study from Utrecht University examined the effect of supplementation with collagen hydrolyzates and a multi-ingredient supplement for 60 days on experimentally induced acute synovitis in horses [50]. Synovitis was induced in the right intercarpal joint by intra-articular injection of 0.5 ng lipopolysaccharide (LPS) of *Escherichia coli*. Although supplementation with collagen hydrolyzates and the combination product showed anti-inflammatory effects in this validated synovitis model and prostaglandin E_2_ (PGE_2_) levels were reduced compared with placebo, no statistical differences were seen with respect to interleukin 6 (IL-6), glycosaminoglycans (GAGs), the biomarker CPII, or matrix metalloproteinases (MMPs) among treatment groups.

A supplement called PETAGILE, which provides collagen peptides, was orally administered to horses with mild or moderate OA for 3 months with daily doses of 25 g and 50 g. A weekly questionnaire to horse owners was provided in order to follow the progress of horse behavior and willingness to run. All 28 horses, (16 received 25 g/day and 12 received 50 g/day of PENTAGILE), improved their mobility and showed increased willingness to run when compared to the placebo group. The higher dosage (50 g) supplementation was concluded to be more effective and promising enough to further test in longer term studies [51].

Clark and colleagues performed a 24-week clinical study on the use of collagen hydrolyzates as a dietary supplement in 147 healthy athletes with activity-related joint pain who were physically fit, active, and had no evidence of joint disease [52]. The study design involved 72 males and 75 females randomly assigned to two groups. The experimental group (*n* = 73) received 25 mL of a liquid formulation of 10 g of collagen hydrolyzates. The placebo group (*n* = 74) received 25 mL of liquid xanthan. The primary measured outcome was a change in the visual analog scales (assessed by a physician) from baseline during the study phase in relation to pain, mobility, and inflammation. The team investigated joint pain at rest and when walking, standing, carrying objects, and lifting. This was the first clinical trial that used a healthy population as a study group and showed improvement in joint discomfort and pain in the group given an oral supplement containing collagen hydrolyzates. Despite the small sample size and limitations of the study, the results suggested that athletes perceived a benefit from consuming collagen hydrolyzates [52].

A clinical study in 15 healthy male subjects was carried out to determine and compare the plasma concentrations of four representative amino acids from collagen (glycine, proline, hydroxyproline, and hydroxylysine) following a single administration of a fresh fermented milk product containing hydrolyzed collagen [53]. This was a single-center, randomized open crossover study. In a fasting state, the 15 healthy subjects randomly received a single dose of product 1 (10 g of collagen hydrolyzate in 100 mL of milk) or product 2 (10 g of collagen hydrolyzate dissolved in 100 mL of water). The study showed that consumption of milk containing collagen hydrolyzate increased the concentration of collagen-specific amino acids in plasma. This suggests that orally ingested collagen hydrolyzates might increase the plasma concentrations of collagen-derived amino acids that could potentially reach tissues in the synovial joint.

Another clinical trial used a randomized double-blind, controlled study design and recruited 250 subjects with primary knee OA to assess the efficacy of a collagen hydrolyzate supplementation on OA pain and function [54]. The patients were given 10 g collagen hydrolyzate daily for 6 months. The authors reported a significant improvement in knee joint function and pain as assessed by visual analog scales as well as the the Western Ontario and McMaster Universities Osteoarthritis Index (WOMAC) pain subscales. Subjects with the greatest joint deterioration, and with the lowest intake of meat protein in their diets, appeared to benefit the most. The study concluded that collagen hydrolyzates are safe and effective and warrant further consideration as a functional food ingredient [54].

McAlindon and colleagues performed biochemical and imaging studies to examine the effect of collagen supplementation in human patients with OA. They attempted to determine whether either of two MRI approaches, delayed gadolinium enhanced magnetic resonance imaging of cartilage (dGEMRIC), or T2 mapping, might detect short-term changes in knee cartilage among individuals taking a formulation of collagen hydrolyzates. Their early results suggest that the dGEMRIC MRI technique may be able to detect changes in proteoglycan content in knee cartilage in individuals taking collagen hydrolyzate after 24 weeks compared to placebo [55]. Only weak correlations were observed between changes in dGEMRIC and biochemical markers, suggesting that the study duration was insufficient to detect measurable changes in biomarkers or that the biomarkers they selected were insufficiently sensitive and discriminatory. They found a positive effect of collagen hydrolyzates on cartilage morphology in patients with knee OA in their interventional OA study (ClinicalTrials.gov: NCT00536302) [56]. However, they could not identify any consistent correlations in changes in collagen and proteoglycan biomarkers PIIANP and CS846 with changes of the dGEMRIC scores in patients who had received oral collagen hydrolyzates. An important weakness of this study was that the authors were looking for short-term changes which are difficult to detect, even after 48 weeks of oral treatment with collagen hydrolyzates. Although the study was time limited, the dGEMRIC score increased in the medial and lateral tibial regions of the knee joint in participants who were given collagen hydrolyzate compared to placebo.

A systematic review published in 2012 examined the evidence on the symptomatic and chondroprotective effects of collagen derivatives in OA. They reported that there is insufficient evidence to recommend the generalized use of collagen hydrolyzates in daily practice for the treatment of patients with OA [57]. They proposed that the overall quality of evidence was moderate to very poor and recommended more independent and high-quality studies to assess the proposed therapeutic effects of collagen derivatives on OA. It is important to note that they did not include studies on only collagen hydrolyzates; they reviewed the evidence from eight different studies: six on collagen hydrolyzates, two on gelatin, and one on undenatured type II collagen (UC-II). As previously mentioned, processing of collagen and their hydrolyzates may result in formulations with differing peptide and amino acid profiles, which may affect patient outcome.

A study published in 2017 examined the metabolic responses of human OA cartilage to biochemically characterized and fractionated collagen hydrolyzates [58]. It compared three different collagen hydrolyzates, two from fish (Peptan® F 5000, Peptan® F 2000) and one from pigs (Mobiforte®) and used biochemical (fluorescence) and biophysical techniques to characterize the products and their effects on human OA cartilage. It also determined the total number of peptides within each product and the peptides that were common between them. The investigators found that none of the three collagen hydrolyzates had the ability to positively modulate collagen biosynthesis in human knee cartilage explants. The authors noted that Peptan® F 2000 enhanced the activities of aggrecanases ADMATS4 and ADMATS5 in vitro. Furthermore, IL-6, MMP-1, -3, and -13 levels were elevated in explants that were treated with Mobiforte® and Peptan® F 5000. This study concluded that due to the heterogeneous peptide composition and disparate pharmacological effects among different collagen hydrolyzates, the effect of a particular preparation or processing cannot be extrapolated to other formulations.

Although there are promising collagen hydrolyzates, the literature has been riddled with poorly designed and executed studies decreasing the credibility of published material, even though positive effects have been demonstrated. For example, another poor study on fish collagen hydrolyzates was published by a group based in Thailand. This group claimed that collagen hydrolyzates can modulate cartilage metabolism [59]. Despite being published, the study was fundamentally flawed because the authors only looked at the effects of collagen hydrolyzates on cartilage explants. Knowing that the explant model is not always suitable for mechanistic studies of hydrolysate action, the results from this study should be interpreted with caution.

The most recent systematic review and meta-analysis of dietary and botanical supplements for OA looked at the evidence supporting the use of collagen hydrolyzates define and UC-II. Although their analysis showed significant improvements on pain, the quality of the published evidence was low and thus the clinical studies were deemed to have limited clinical impact [19••]. The poor quality of published literature highlights the many knowledge gaps regarding collagen-based nutraceuticals, which require further high-powered and well-designed studies to propose evidence-based recommendations.

## Emerging Research for Collagen Hydrolyzates

**Fig. 3:**
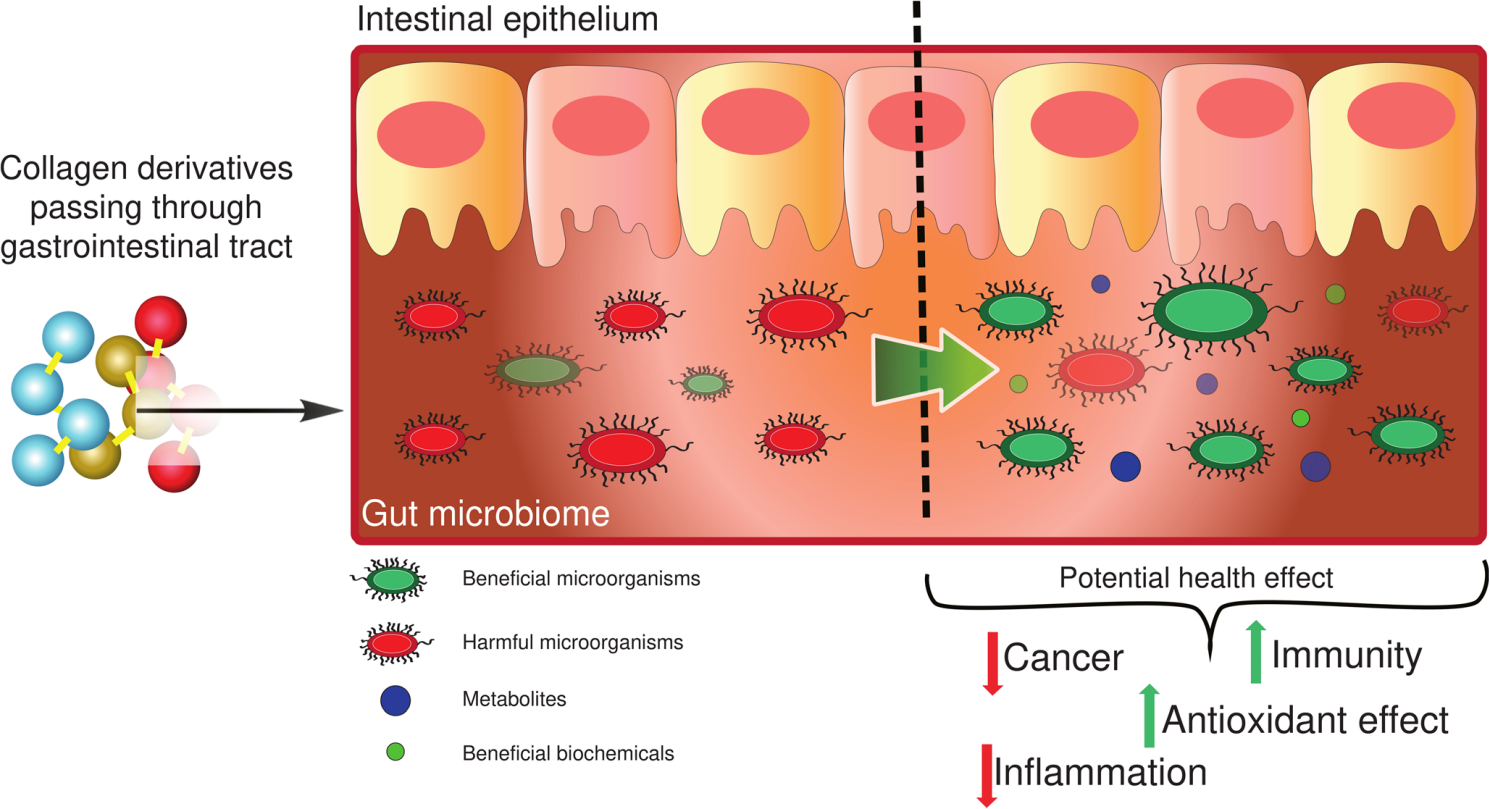
Possible mechanism of pre- or pre-biotic modulation by collagen derivatives

Research into collagen hydrolyzates has primarily focused on the benefits these products can have on joints, most often on cartilage tissue and subchondral bone. However, new and emerging fields of research have shown promise regarding the additional health benefits these supplements can provide, due to their significant peptide and amino acid content and their general tolerability. A key new direction of the field is in the study of how collagen hydrolyzates support biological effects that are relevant in OA, with an emerging interest in how these supplements may modulate the gut microbiome by acting as potential pre-biotics (Fig. 3). The classical definition of a prebiotic is a food component that can change the activity or growth of the microorganisms found in the gastrointestinal tract. Changing the growth of different members of the microbiome can have implications in human health via modulation of the immune system and the production of metabolic byproducts of the gut flora that have biological action in the host.

The gut microbiome is the community of bacteria that resides in the gastrointestinal tract, includes the metabolic byproducts produced by the resident microbes. The amount of bacteria in and on the human body is far greater than the number of eukaryotic cells. The microbiome has a substantial impact on human health and clinical outcomes. The gut microbiome has been shown to affect multiple physiological pathways and disease states, including colon cancer, amyotrophic lateral sclerosis, RA, type 2 diabetes, metabolic syndrome (MetS), and Alzheimer’s disease. It is implicated in numerous inflammatory gastrointestinal diseases such as inflammatory bowel disease (IBD) and irritable bowel syndrome, and it is known to influence various musculoskeletal diseases, OA, and osteoporosis (OP) [60–65]. A recent study by Schott et al. (2018) investigated the role of the gut microbiome in the context of obesity-associated OA [66]. Obesity was induced using a high-fat diet with mice that were given a lean diet as a control. OA was induced using destabilization of the medial meniscus surgery. Mice were then supplemented either with oligofructose, a nondigestible prebiotic fiber, or cellulose, a control fiber. Results demonstrated that the gut microbiome of obese mice reverted to a state similar to that of the lean control mice after prebiotic supplementation. In the context of obesity, oligofructose supported the growth of key microflora, particularly *Bifidobacterium pseudolongum*. Furthermore, obese mice treated with oligofructose demonstrated cartilage preservation and an increased number of chondrocytes in the tibia and femur, as well as decreased OARSI scoring. No histological differences were observed in mice fed a low fat diet with or without prebiotic supplementation. Both systemic and synovial inflammation in obese mice were also reduced by oligofructose supplementation and the consequent changes in the microbiome. This report provided the first strong evidence for a link between the gut microbiome and OA [66]. This connection between the microbiome and joint health may also explain why some individuals respond to collagen hydrolyzate supplements while others do not. Interindividual differences in digestion and absorption rate of collagen hydrolyzate peptides and amino acids could also influence a patient’s response.

Perhaps the most intriguing explanation for interindividual differences in the digestion, uptake, and efficacy of collagen hydrolyzates and nutraceuticals in general is the substantial variability between the microbiomes of different individuals. Countless intrinsic and extrinsic factors, including diet, genetics, exercise level, diurnal rhythm, and even time of day contribute to different microbiome states within the niche of an individual gastrointestinal tract. Even when these factors are controlled, as in animal studies using defined diet, light cycle, and age matched controls, the specific microbiome fingerprint remains different between individual animals.

In a recent mouse study of gut microbiome, OA, and nutraceuticals, different microbiome profiles resulted from consumption of standard chow, high fat, and lean diets, and overlay of supplementation with UC-II or GS led to significant shifts in the constituent microbes [67]. Although the microbiome of each animal was distinct from all other animals, clear types were produced by the different supplements, with *Veillonella*, *Bifidobacteria*, and *Ruminococcus* genera contributing substantially to these profiles.

It is unsurprising that defined diet influences both the gut microbiome and OA outcomes in mice. What is intriguing is that dietary supplementation with nutraceuticals like chondroitin sulfate (CS) causes shifts in the gut microbiome within the same control diet in mice [68]. In these animals, CS supplementation resulted in butyrogenic changes in the microbiome metabolism and lowered inflammatory LPS levels in circulation and caused a concomitant decrease in proinflammatory taxa and increase in anti-inflammatory taxa. Because OA has been shown to be a disease of inflammation, it is possible that the action of CS on OA is via the gut microbiome.

In human metabolism, degradation of CS is both variable and dictated in part by the innate gut microbiome. A 2016 study found that CS breakdown was caused by three different *Bacteroides* species in the gut found in all human participants. Even more interestingly, individuals in the study possessed at least two taxa that were not found in other study members that could also degrade CS [69]. It appears that the gut microbiome contributes to the metabolism of CS, within different population groups as well as between individual members of the groups; based on this it is not surprising that nutraceutical response is variable in the published literature.

In a study examining microbiome and OA changes caused by dietary supplementation with either GS or UC-II, a distinct microbiome profile was found for each group. A low Bacteroidetes/Firmicutes ratio has been associated with a proinflammatory state in both mice and humans [70, 71]. Compared to mice that were not given a nutraceutical, supplementation with either GS or UC-II resulted in a higher Bacteroidetes/Firmicutes ratio [67]. Individual operational taxonomic units (OTUs) were different in animals given nutraceuticals compared to animals given control fibers; furthermore, OTUs were significantly different depending on whether GS or UC-II was provided. Overall, the study found that supplementation with either GS or UC-II resulted in OTU changes that could be associated with lowered inflammatory states; ingestion of UC-II caused more changes in OTU abundances compared to GS, but there were nutraceutical specific microbiome changes in the case of GS and *Rikenellaceae*. Perhaps most crucially, dietary supplementation with either nutraceutical increased tibial total and uncalcified cartilage area, as well as Safranin O chondrocytes, compared to the control fiber, in a post-traumatic OA (PTOA) model. Collectively, these data implicate the gut microbiome as a mediator of nutraceutical action on OA outcomes.

Because of the microbiome and gastrointestinal tract relevance to nutraceuticals and OA, future collagen and OA research needs to implement the latest gastrointestinal models; these need to be scalable, fast, and physiologically relevant. Initiatives to make these digestion models more affordable and accessible have started [72–74]. Future use of computer a controlled dynamic digestion model inoculated with human fecal matter can be used to investigate the digestibility of collagen hydrolyzates and their effect on the microbiome. In such bioreactor models, the stomach, small intestine, and three colonic vessels (ascending, transverse, and descending) are continuously agitated, pH controlled, and the digesta are propelled along the simulated gut model using peristaltic pumps. As our understanding of the microbiome and its impacts on overall health and OA evolve, simulated gut model studies investigating supplements that are readily available to patients are required to determine both the beneficial and potentially deleterious effects these products may have on the gastrointestinal system and how they affect OA.

Gastrointestinal models provide a unique opportunity to test collagen hydrolyzate products, as well as other drugs. These models can be inoculated with different human gut microbiota, allowing the impact of nutraceuticals and newly developed drugs on the gastrointestinal tract to be investigated. These models could also utilize the microbiota of patients with varying degrees of OA, first to determine if the microbiome between OA patients at different stages are different, and then to investigate the impact of new treatments. This model could function as a pre-clinical tool where the safety and toxicity of new drugs and nutraceuticals can be determined.

## Challenges of Research with Nutraceuticals

### Regulations

Due to the “non-medical” origin of nutraceuticals, there is a lack of regulatory methods and proof of efficacy requirements in Europe and the USA. But like pharmaceutical products, nutraceuticals should require strict regulations and evidence-based research that confirm efficacy, safety, and benefits to the patient [14].

### Measurement Control

OA is lacking an established measurement–control system for an objective evaluation of pre-clinical changes potentially indicating an important windows of treatment opportunity. Recognizing OA in the early pre-OA or early OA phase is increasingly accepted as being such a window of opportunity. MRI has the capacity to diagnose OA in this early phase and DMOADs developed by Merck and Novartis have used this imaging technique for the assessment of structural progression [75]. For DMOADs, both functional and structural outcomes are considered mandatory by the FDA. Next to MRI, biochemical markers measured in synovial fluid, but preferably non-invasively, from urine, or from plasma or serum may aid the timing, registries and database building followed by outcome measurement and algorithm building to support joint-preserving treatments for OA in the field of lifestyle/nutrition, pharmacological interventions, and joint-preserving surgery. Ideally, such a measurement–control system is species independent to translate the same outcome measures from animal studies to clinical implementation. In this light, developments in the area of high-field MRI, mass spectrometry, and a combination thereof, are highly promising.

### Animal Studies

Animal studies are generally slow, costly and predictions of bio-absorbability do not always align with human clinical data owing due to species differences in intestinal permeability and metabolic activity [76, 77]. The bioavailability of food components is determined by first-pass metabolism, which involves absorption by enterocytes found in the gastrointestinal tract, followed by liver metabolism before entering the systemic circulation [78, 79]. Bioavailability studies of food components and pharmaceuticals using animal models have previously established poor correlation between rats and humans. Due to these species differences in intestinal permeability and metabolic activity, in vitro digestion and cell culture models, rather than animal models, are often used to assess the digestion profile and intestinal transport of orally administered food components or drugs. In vitro digestion models are often used to assess for nutrient digestion before first pass metabolism, as human trials are difficult and impractical for routine nutrient bioavailability assessments [80–82]. Previous and ongoing validation studies continue to support the use of in vitro digestion models for testing nutrient digestion, and for bioactive peptides, by comparing to in vivo results [82, 83]. One of the limitations of previous research on collagen products is that they have often used these products directly on tissues, such as cartilage, to determine their effects, but in a physiological context, these products first undergo digestion and first pass metabolism. Future research needs to consider more holistic and physiologically relevant approaches to validate mechanisms of action of collagen-based products for the treatment of OA.

### Human studies

Human studies provide the most accurate and valuable outcomes, although there are still several limitations and study design choices which should be taken into consideration. One of these parameters is the choice of test and control groups, which should be distinguishable from one another, and the selection of either of these groups must be based on specific demographic and population variables. For example, as nutritional requirements differ according to a person’s age, the target population of a nutraceutical should be focused on a specific age group. If the target population is the elderly, difficulties arise as it is challenging to identify all risk factors, comorbidities, and possible interactions, as well as to model a study according to all these aspects. The exposure to nutraceuticals and the interindividual variation of response are also crucial aspects, as well as the fact that nutraceuticals are subjected to intestinal bacterial metabolism which can generate active or inactive metabolites. Capping off the complexity are considerations around disease etiology. The OA syndrome is not driven by a single pathogenic mechanism, but rather can be initiated by various factors including age, obesity, genetics, and injury. Specific supplements may have efficacy in one context but not another, and so studies should consider information about likely initiators of degeneration. To understand the effects of a nutraceutical with these variables in mind, studies with large populations and elevated financial expense are necessary [84]. Other crucial features of a study to be considered are duration, timing, and budget.

The formulation in which a nutraceutical is manufactured can also have an impact related to the pharmacokinetics of the relevant bioactives in terms of absorption, distribution, metabolism, and excretion (ADME). The ADME can determine factors such as dose, half-life, and frequency of intake of the supplement that impact bioactivities. Formulation may not necessarily cause adverse effects or be harmful to therapeutic outcomes but could alter the absorption rate and efficacy of the nutraceutical. Formulation should be chosen according to the origin of the functional elements included in the preparation, its solubility, resistance to pH changes, and impact of shifts in the microbiota on permeability and stability of the nutraceutical. Novel Drug Delivery Systems (NDDS) compared to simple and widely used formulations offer advantages by enhancing stability, providing sustained release, and protecting the compound(s) from physical or chemical degradation [85].

Many challenges surround the evaluation of nutraceutical efficacy, safety, and regulations. As interest and consumer consumption increases, the lack of clear clinical evidence is still a limitation. To address this and ensure the best outcomes for consumers, a variety of complementary research approaches are needed. In that regard, in vitro studies can be used to provide proof of concept, for safety assessment, and to help understand how a supplement might impact on structure and function. Appropriate animal models for OA can also aid in assessments of nutraceuticals in terms of potency, safety, and mechanisms of action. These studies can evaluate the impact of both acute and long-term nutraceutical supplementation and control for confounding variables such as genetics, sex, and background diet. In vitro and in vivo preclinical studies aid the determination of relevant biomarkers, outcome measures, and the experimental design for human nutraceutical studies, which are crucial for validation. Clinical studies remain the most verifiable approach to evaluate how interindividual variabilities in gut microbiota and host response involving differing genetic, biochemical, and anatomical characteristics can impact the health promoting properties of nutraceuticals. It should be recognized, however, that the complexity of nutraceutical metabolism leads to a large variety of metabolites that make it unlikely to characterize all pertinent features of metabolic activity. Moreover, a wide variety of baseline lifestyle factors including nutritional status, and background dietary and exercise habits could lead to variable interindividual responses to nutraceutical supplementation.

## Future Innovations in Nutraceuticals

By 2025, the collagen market is estimated to be valued at $6.63 billion, and in this year alone, U.S. consumers are expected to spend around $122 million on collagen products. The largest component will be cosmetic collagen, but a substantial portion of the emerging market will be collagen-based nutraceuticals for bones and joints. Patients with OA consume supplements along with OTC products [11]; they hope for readily available, new, and innovative nutraceuticals. There is an opportunity to develop combination nutraceuticals incorporating collagen ultra-hydrolyzates since supplements remain very appealing for many patients dissatisfied with current conventional drugs.

## Conclusions

A growing body of work has accumulated to provide a scientific rationale for the use of oral collagen hydrolyzates to treat patients with OA. However, evidence for their clinical efficacy is lacking and mechanistic and targeted clinical research is required to determine if and how collagen hydrolyzates may help to improve joint health [86, 87]. We need to determine which OA phenotypes and subpopulations are the most appropriate for demonstrating the potential benefits of oral collagen supplements. Collagen hydrolyzates have demonstrated some evidence of efficacy in several small scale clinical trials, but more research is needed. Collagen hydrolyzates are likely to have a much greater impact in patients with early OA compared to patients with advanced OA. Also, collagen hydrolyzates have the potential for use in a healthy population without OA, as a preventive and prophylactic measure. Collagen hydrolyzates are considered an attractive nutritional supplement for preventing bone and joint degeneration in early stages of OA, but their ability to treat and reverse advanced joint disease remains highly speculative. Novel and innovative research continues to be published on collagen hydrolyzates and links with the microbiome, but more work is needed. We advocate new interdisciplinary collaborative initiatives, at the academic and industrial level, to develop new products and critically evaluate the impact of collagen-based nutraceutical supplements for patients with OA and related osteoarticular disorders.

## Abbreviations

ACR: American College of Rheumatology
ADME: Absorption, distribution, metabolism, and excretion
AF: Arthritis Foundation
BCFA: Branched chain fatty acid
CS: Chondroitin Sulfate
dGEMRIC: delayed gadolinium enhanced magnetic resonance imaging of cartilage
DMOAD: Disease modifying OA drug
DSHEA: Dietary Supplement Health and Education Act
EC: European Commission
ECM: Extracellular matrix
ESCEO: European Society for Clinical and Economic Aspects of Osteoporosis, Osteoarthritis and Musculoskeletal Diseases
EU: European Union
EULAR: European League Against Rheumatism
FDA: Food and Drug Administration of the United States
GAGs: Glycosaminoglycans
GS: Glucosamine sulfate
IL-6: Interleukin 6
IBD: Inflammatory bowel disease
LPS: Lipopolysaccharide
MetS: Metabolic syndrome
MMP: Matrix metalloproteinase
MRI: Magnetic resonance imaging
MW: Molecular weight
NDDS: Novel Drug Delivery Systems
OARSI: Osteoarthritis Research Society International
OA: Osteoarthritis
OP: Osteoporosis
OTC: Over the Counter
OTUs: Operational taxonomic units
PGE_2_: Prostaglandin E_2_
PTOA: Post-traumatic OA
RA: Rheumatoid arthritis
RMDs: Rheumatic and musculoskeletal diseases
SCFA: Short chain fatty acids
SYSADOAs: Symptomatic slow-acting drugs for OA
UC-II: Undenatured Type II Collagen
UN: United Nations
WOMAC: Western Ontario and McMaster Universities Osteoarthritis Index
